# The value of caudate lobectomy in hilar cholangiocarcinoma treatment: A meta-analysis

**DOI:** 10.1097/MD.0000000000024727

**Published:** 2021-02-19

**Authors:** Ming Yang, Wei Wei Li, Jian Hua Chen, Miao Hang Cui, Jin Long Liu

**Affiliations:** a Department of hepatobiliary surgery, The Affiliated Hospital of Chengde Medical College; b Department of General Surgery, Kuancheng County Traditional Chinese Medicine Hospital, Chengde City, Hebei Province, China.

**Keywords:** caudate lobectomy, hilar cholangiocarcinoma, meta-analysis, partial hepatectomy, prognosis

## Abstract

**Purpose:**

To discuss the value of caudate lobectomy in hilar cholangiocarcinoma (HCCA) treatment.

**Methods:**

A systematic review was performed in PubMed, MEDLINE database, EMBASE, and Cochrane Library for trials comparing combined caudate lobectomy with controls from January 1, 1990 to December 2, 2020. The outcomes were postoperative radical cure information, survival condition, morbidity, and mortality.

**Result:**

Ten studies were included. No difference was observed in the morbidity (odd ratio (OR) 0.93, 95% confidence interval (CI) 0.65–1.33) and mortality (OR 1.16, 95% CI 0.55–2.42) between the combined caudate lobectomy and control groups. Hepatectomy combined with caudate lobectomy was associated with higher incidence of radical resection (OR 3.88, 95% CI 2.18–6.90) and longer survival (hazard ratio 0.45, 95% CI 0.38–0.55).

**Conclusion:**

Combining caudate lobectomy can significantly increase the incidence of radical resection of HCCA and the postoperative survival time. The morbidity and mortality were not increased after the operation. Thus, caudate lobectomy should be included when performing partial hepatectomy for HCCA.

## 1 Introduction

Hilar cholangiocarcinoma (HCCA) is a type of bile duct cancer arising from the biliary confluence. For a decade, the incidence of HCCA has been increasing and it accounts for about 50%–60% of all biliary tumors; these patients usually have a poor prognosis.^[1–3]^ Radical resection of the tumor plays a key role in extending the overall survival.^[4,5]^ HCCA is inclined to invade the hepatic parenchyma along the bile duct, whereas the bile duct of the caudate lobe is connected to the hepatic duct in the hilar portion. The anatomical characteristic increases the risk of tumor encroaching on the caudate lobe. There has been a heated discussion about the need to remove the caudate lobe that may be invaded.

Due to the low incidence of HCCA and great difficulty in treatment, there is still a lack of large sample-sized studies on the postoperative radical effect, long-term prognosis, postoperative complications, and surgical death due to caudate lobectomy for HCCA. In the surgical treatment of HCCA, does the inclusion of caudate lobe resection improve the postoperative radical cure rate and prolong the postoperative survival? Does the trauma increase the risk of postoperative complications and mortality? These problems remain to be solved. The purpose of this study was to explore the effects of caudate lobe resection for HCCA by examining the literature, so as to provide medical evidence for the surgical treatment of HCCA.

## 2 Methodology

### 2.1 Ethical statement

This work was based on previously published studies. Therefore, no ethical approval or patient consent was necessary.

### 2.2 Literature search

Studies were identified via an electronic search of PubMed, MEDLINE database, EMBASE, and Cochrane Library using the following retrieval formula ((hilar cholangiocarcinoma) or (Klatskin tumor)) and ((caudate lobe) or (caudate lobectomy)). The search covered from January 1, 1990, to December 2, 2020. The language of publication was restricted to English. The references of articles and reviews were manually searched for additional studies. We also hand-searched the journals that published articles most relevant to this review.

### 2.3 Inclusion and exclusion criteria

The systematic review generated complete databases from published studies dealing with the prognosis of patients with HCCA treated by caudate lobectomy or not. The language of publication was restricted to English. To be eligible for inclusion, studies had to meet the following criteria:

(1) object of the studies was patients with HCCA;
(2) all of the patients received surgical treatment;
(3) the literature analyzed the prognosis of patients with HCCA who underwent caudate lobectomy or not; the prognosis included at least one of the following four indexes: radical resection, long-term survival, postoperative complications, and short-term mortality;
(4) they contained a hazard ratio (HR) and 95% confidence interval (CI) for survival according to surviving status, which were either reported or could be computed from the data presented;
(5) when the same author or group reported results obtained from the same patient population in more than one article, the most recent report or the most informative report was included; and
(6) the study quality was evaluated as higher than 5 stars according to the Newcastle–Ottawa quality assessment scale.^[6]^

The exclusion criteria were as follows:

(1) the prognostic effect was valued by the recurrence rate of the patients;
(2) letters, reviews, case reports, conference abstracts, editorials, and expert opinion were excluded; and
(3) the prognosis of single surgical treatment was reported without a control group.

### 2.4 Data extraction

Two investigators (LJL and YM) reviewed all of the research that met the inclusion and exclusion criteria. Data were extracted independently by 2 investigators (LJL and YM) using a data extraction sheet. Data extracted included the first author's name, year of publication, source of patients, number of patients, combined caudate lobectomy or not, number of radical resection procedures, survival data (HR and 95% CI), number of postoperative complications, and postoperative short-term mortality. If the data were controversial, 2 data extractors jointly resolved the problem.

### 2.5 Assessment of study quality

Study quality was assessed independently by 2 investigators (LJL and CJH) by means of reading and evaluating according to the Newcastle–Ottawa quality assessment scale. Briefly, the overall star system assesses the following three main categories:

(1) selection of the cohort,
(2) comparability of the cohort, and
(3) ascertainment of the outcome.

A study can be awarded a maximum of 1 star for each numbered item within the selection and outcome categories. A maximum of 2 stars can be given for comparability. The total number of stars was counted at the final stage, with more stars reflecting higher methodological quality. A study can be awarded a maximum of nine stars.

### 2.6 Statistical analysis

In the study, odds ratio (OR) and 95% CI were used to evaluate the effect of different surgical procedures on radical resection, postoperative complications, and short-term mortality of patients with HCCA.

HR and 95% CI were used to estimate the impact of different surgical procedures on survival. If the HR in the literature was less than 1, the survival time in the combined caudate lobectomy group was longer than that in the caudate lobe preserved group. Otherwise, the HR value was converted by the formula of LN (HR).

If a direct report of HR and 95% CI was not available, the estimated value was derived indirectly from Kaplan–Meier curves using the methods described by Tierney.^[7]^ Kaplan–Meier curves were read by Engauge Digitizer version 4.1 (http://digitizer.sourceforge.net/), and then the survival data read from Kaplan–Meier curves were entered into the spreadsheet appended to Tierney's paper.^[7]^ This work was performed by 2 independent persons to reduce inaccuracy in the extracted survival rates.

To assess the heterogeneity among the studies, we used the Cochran Q and I^2^ statistics. For the Q statistic, a *P* value < .10 was considered statistically significant for heterogeneity.^[8]^ Then the random effects model was calculated according to the DerSimonian-Laird method.^[9]^ Otherwise, the fixed-effects model (Mantel–Haenszel method) was used. For I^2^, a value > 50% was considered a measure of severe heterogeneity;^[10]^ thus, the conclusion was derived with discretion, or the combination of HRs was given up. The funnel plot and Egger test were used to evaluate publication bias in the caudate lobectomy survival group. To test the robustness of the conclusions obtained from the meta-analysis of the caudate lobectomy survival group, sensitivity analysis was also conducted. All of the statistical analyses were performed by Stata 12.0 (Stata Corporation, College Station, Texas, USA). A significant 2-way *P* value for comparison was defined as *P* < .05.

## 3 Results

### 3.1 Literature selection

**Figure 1:**
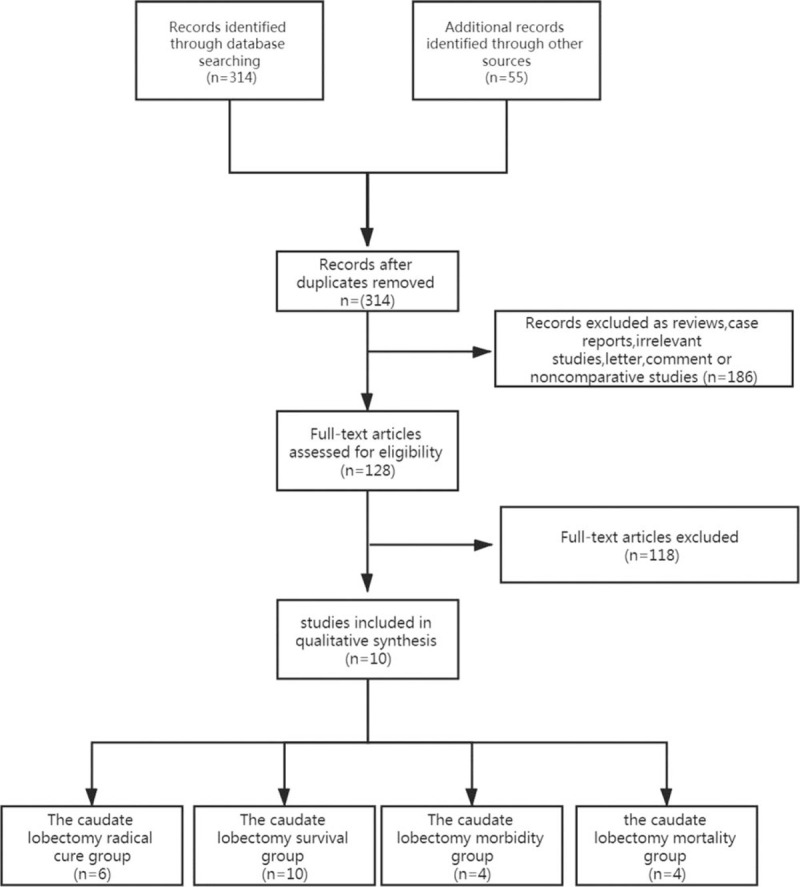
Flow diagram of study selection.

A total of 314 potentially relevant citations were retrieved after the initial search of databases. Although another 55 studies were found from the references of articles and reviews or by hand-search of the journals, all of them were duplicates of studies from the database search. The title and abstract of relevant articles were read by the 2 authors (LJL and CJH) independently. One hundred and eighty-six were excluded from the analysis after the first screening based on abstracts or titles, leaving 128 articles available for further full-text review. After carefully reading the full-text articles, 118 studies were excluded. The final 10 studies were in line with the inclusion criteria.^[11–32]^ Ten studies satisfied the inclusion criteria, and they belonged to the following four study groups:

- the caudate lobectomy radical cure group (6 papers),^[14,29,33–36]^
- the caudate lobectomy survival group (10 papers),^[11,14,29,33–39]^
- the caudate lobectomy morbidity group (4 papers),^[33–36]^ and
- the caudate lobectomy mortality group (4 papers).^[33–36]^ (Fig. 1)

### 3.2 Methodological quality of the studies

For the included studies, 2 authors independently extracted data and assessed the methodological quality using the Newcastle–Ottawa quality assessment scale. The scores are shown in Table 1. All of the studies included in our meta-analysis had high levels of methodological quality (> 5 stars on the Newcastle–Ottawa scale).

**Table 1:** Characteristics of included studies and the corresponding study groups.

First author	Newcastle–Ottawa Score	Publish yr	Country	Study group
Cho, M. S.^[14]^	9	2012	South Korea	1,2
Song, S. C.^[29]^	9	2013	South Korea	1,2
Aj, I. Jitsma^[37]^	8	2004	Netherlands	2
Cheng, Q. B.^[34]^	7	2012	China	1,2,3,4
Kow, A. W.^[35]^	9	2012	South Korea	1,2,3,4
Wahab, M. A.^[36]^	8	2012	Egypt	1,2,3,4
Zheng-Rong, L.^[39]^	7	2011	China	2
Gazzaniga, G. M.^[38]^	7	2000	Italy	2
Bhutiani, Neal.^[33]^	7	2018	United States of America	1,2,3,4
Abd ElWahab^[11]^	8	2016	Egypt	1,2

### 3.3 Indications of caudate lobectomy in the included studies

The decision to perform caudate lobectomy was based on surgeon's experience^[11,14,29,33–36,38,39]^ and the results of frozen section of resection margin.^[37]^

### 3.4 Assessment of heterogeneity

The Q test and I^2^ test were used to assess the heterogeneity between each study group. We found that there was no significant heterogeneity in the caudate lobectomy survival group (I^2^ = 38.2%, *P* = .103), the caudate lobectomy morbidity group (I^2^ = 0.0%, *P* = .411), and the caudate lobectomy mortality group (I^2^ = 0.0%, *P* = .568), whereas the heterogeneity in the caudate lobectomy radical cure group (I^2^ = 65.9%, *P* = .012) was significant.

## 4 Results of the meta-analysis

### 4.1 The caudate lobectomy radical cure group

**Figure 2:**
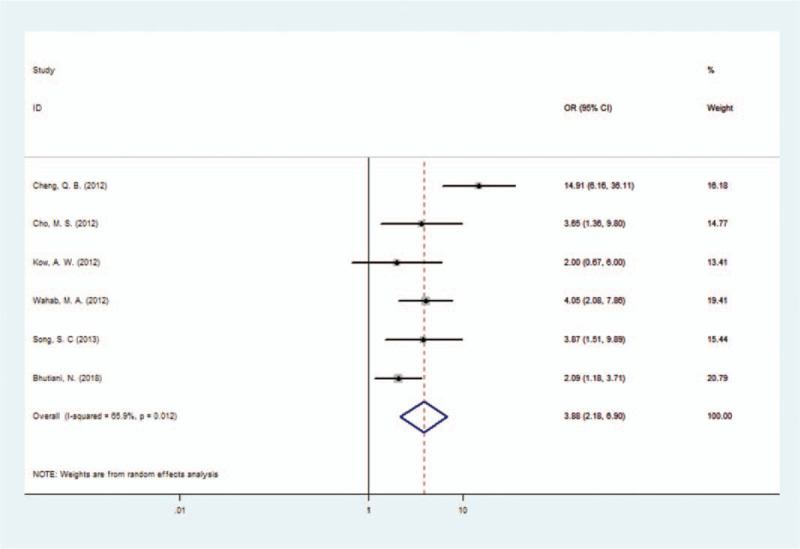
Forest plot of the caudate lobectomy radical cure group.

In the 6 papers included, a total of 986 patients with HCCA were reported, including 540 patients treated with combined caudate lobectomy and 446 patients with caudate lobe preserved. All caudate lobectomies were performed on the basis of left or right hepatectomy. The caudate lobe was preserved with partial hepatectomy in the control group. In the caudate lobectomy radical cure group, the combined radical cure rate was 84.81% (458/540) after combining caudate lobectomy, whereas the combined radical cure rate was 60.31% (269/446) with caudate lobe preserved. The radical resection rate in the combined caudate lobectomy group was significantly higher than that in the caudate lobe preserved group. The combined OR was 3.88 (95% CI: 2.18–6.90). The heterogeneity of the group was significant (I^2^ = 65.9%, *P* = .012), which may affect the accuracy of the conclusion (Fig. 2).

### 4.2 The caudate lobectomy survival group

In the 10 papers included, a total of 1330 patients with HCCA were reported, including 665 patients treated with combined caudate lobectomy and 665 patients with caudate lobe preserved. Caudate lobectomies were performed on the basis of partial left or right lobectomy. The control group included patients with partial hepatectomy but caudate lobe preserved. In the papers included, the three-year survival rate in HCCA patients was reported to range from 36.2%^[39]^ to 43%^[36]^ and the 5-year survival rate was reported to range from 10.6%^[39]^ to 33%.^[29]^ The combined HR of 10 papers suggested that the survival of HCCA patients treated with combined caudate lobectomy was significantly better than that of patients with caudate lobe preserved (HR 0.45,95% CI 0.38–0.55).

**Figure 3:**
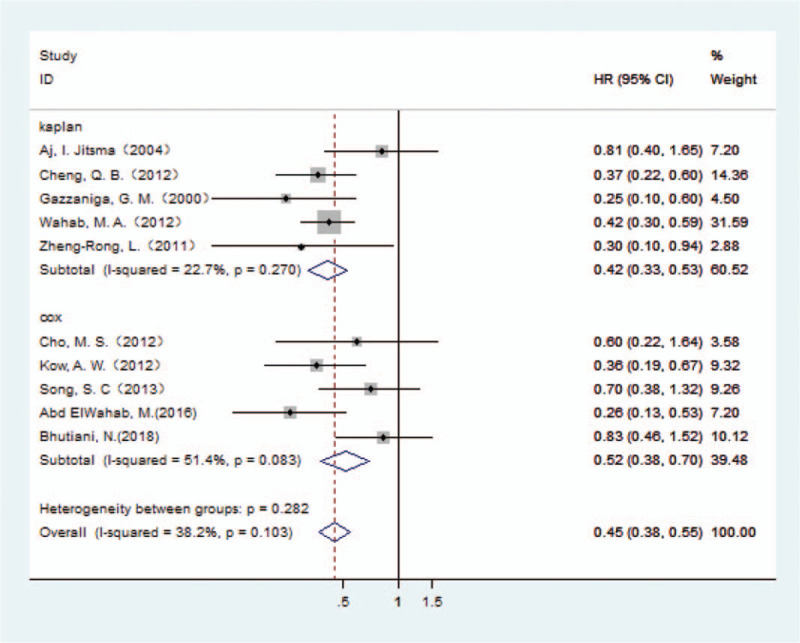
Forest plot of the caudate lobectomy survival group. Subgroup analysis by the source of HR value is shown.

Among the 10 papers, 5 papers^[11,14,29,33,35]^ provided HR values and 95% CI directly. In the other 5 papers,^[34,36–39]^ we obtained the HR value from the Kaplan–Meier curve by Tierney's method.^[7]^ Subgroup analysis was performed according to the source of the HR value. As the *P* value of the Q test was less than .10, the fixed effect model was used. On subgroup analysis, based on whether HR values were derived from direct or indirect calculations, the combined HR values suggested that the survival of patients with HCCA treated with combined caudate lobectomy was significantly better than that of patients with caudate lobe preserved (Fig. 3).

### 4.3 The caudate lobectomy morbidity group

**Figure 4:**
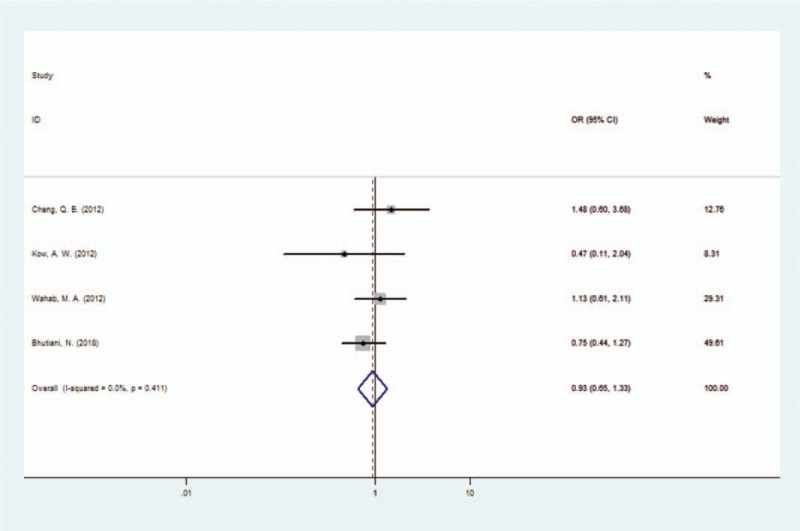
Forest plot of the caudate lobectomy morbidity group.

In the 4 papers included, a total of 713 patients with HCCA were reported; including 377 patients treated with combined caudate lobectomy and 336 patients with caudate lobe preserved. Complications of surgery for HCCA reported in the papers included pneumonia, biliary leakage, intraperitoneal hemorrhage, and incision infection. After combining the surgeries, complications occurred in 36.34% (137/377) of the patients treated with combined caudate lobectomy, whereas 47.92% (161/336) of the patients developed complications in the caudate lobe preserved group. The combined OR suggested that the risk of postoperative complications in the caudate lobectomy group was not higher than that in the caudate lobe preserved group (OR 0.93, 95% CI 0.65–1.33), as shown in Figure 4.

**Figure 5:**
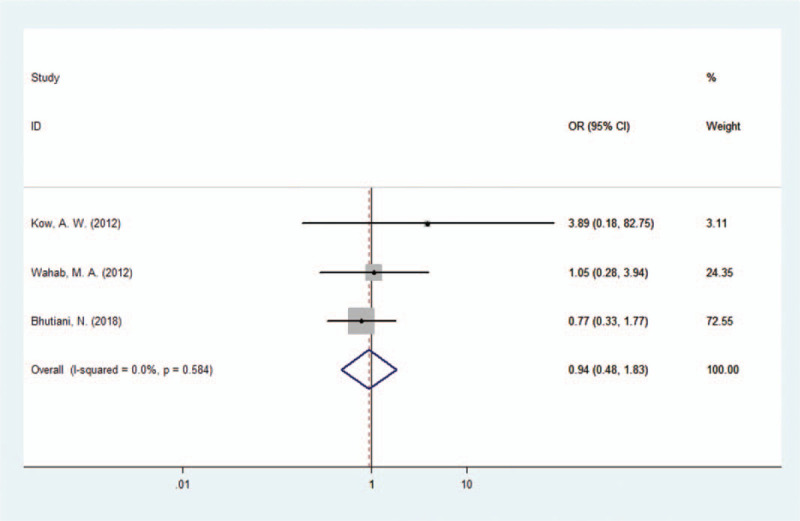
Forest plot of the caudate lobectomy morbidity (total postoperative complications) group.

According to the available information provided in the 3 studies included,^[33,35–36]^ biliary leakage occurred in 7.50% (18/240) of the patients treated with combined caudate lobectomy, whereas 8.94% (27/302) of the patients in the caudate lobe preserved group. The combined OR suggested that the risk of biliary leakage in the caudate lobectomy group was not higher than that in the caudate lobe preserved group (OR 0.94, 95% CI 0.48–1.83), as shown in Figure 5.

### 4.4 The caudate lobectomy mortality group

**Figure 6:**
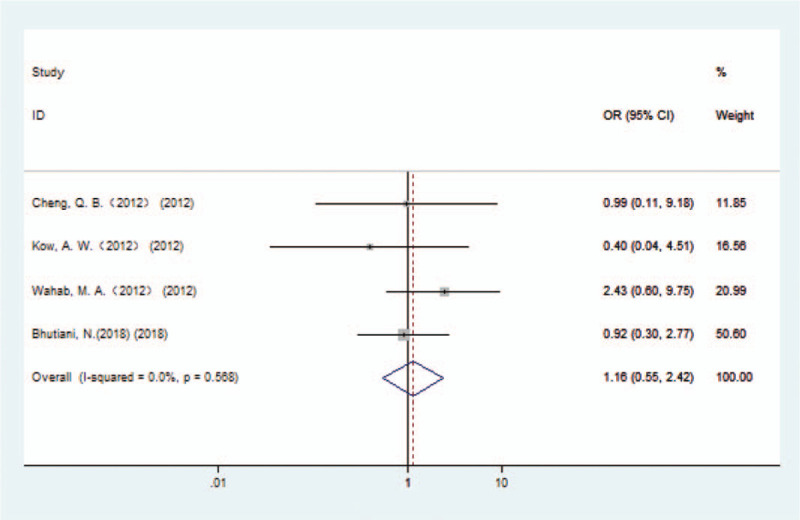
Forest plot of the caudate lobectomy mortality (biliary leakage) group.

In the 4 papers included, a total of 713 patients with HCCA were reported; including 377 patients treated with combined caudate lobectomy and 336 patients with caudate lobe preserved. The reported causes of death in HCCA patients were infections, liver failure, and abdominal bleeding. After caudate lobectomy, 4.51% (17/377) of patients with HCCA died, compared with a rate of 4.76% (16/336) in the caudate lobe preserved group. The combined OR suggested that the risk of postoperative short-term mortality in the combined caudate lobectomy group was not higher than that in the caudate lobe preserved group (OR 1.16, 95% CI 0.55–2.42) (Fig. 6).

### 4.5 Publication bias

**Figure 7:**
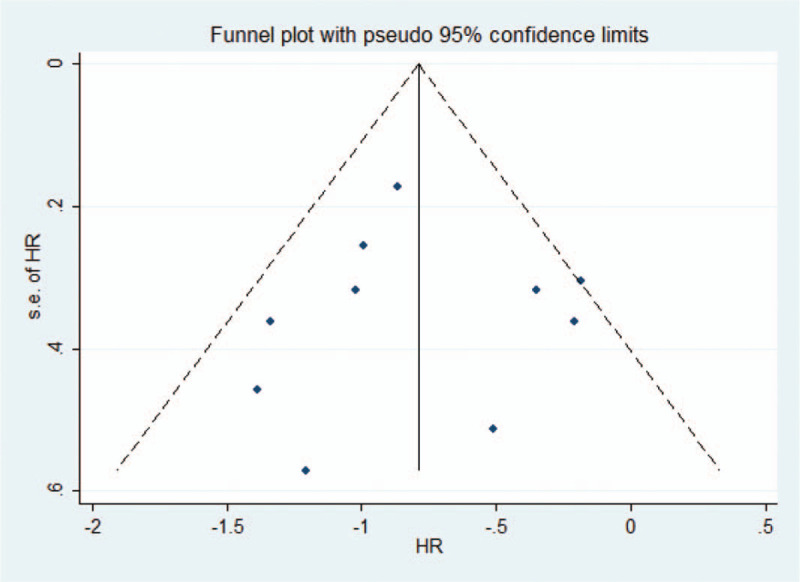
Funnel plots were used to detect publication bias in the caudate lobectomy survival group. Studies are distributed symmetrically, and they suggest that publication bias is absent in the meta-analysis.

To determine whether there was a publication bias in the papers of the caudate lobectomy survival group in this study, a funnel diagram (Fig. 7) was drawn. The symmetry state indicated that there was no publication bias in the research group. We also performed the Egger test by Stata 12.0 (Stata Corporation, College Station, Texas, USA). There was no evidence in the caudate lobectomy survival group (*P* = .894).

### 4.6 Sensitivity analysis

**Figure 8:**
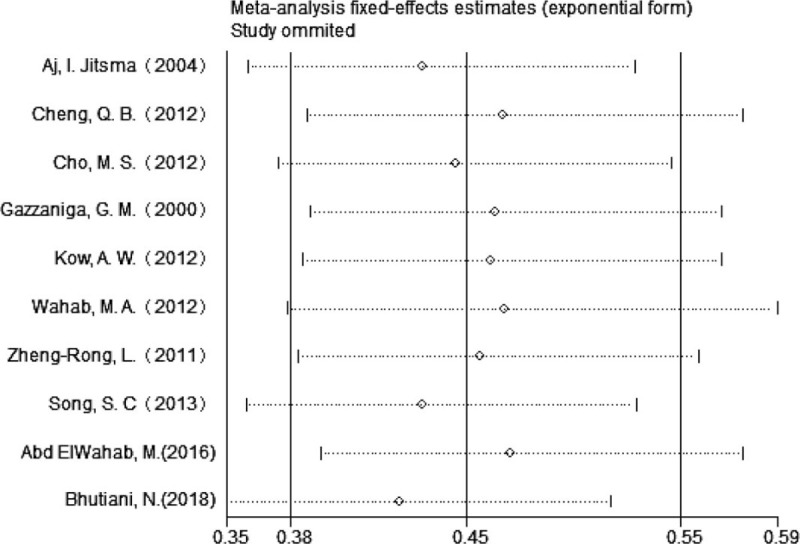
Sensitivity analysis was conducted to test the robustness of the conclusion in the caudate lobectomy survival group.

To test the robustness of the conclusion obtained from the meta- analysis of the caudate lobectomy survival group, sensitivity analysis was conducted (Fig. 8). On the sensitivity analysis, we could see that removing any papers did not affect the combined conclusion of the caudate lobectomy survival group; thus, the conclusion was stable and reliable.

## 5 Discussion

It is debatable whether the caudate lobe should be removed in patients with HCCA. The caudate lobe of the liver is located between the left and right lobes of the liver, and it surrounds the inferior vena cava. The bile duct of the caudate lobe opens in the left or the right hepatic duct (the most common opening is in the left hepatic duct), and HCCA can invade the caudate lobe through the bile duct. The incidence of caudate lobe invasion is reported to range between 31% and 98%.^[40,41]^ It is also found that cancer cells can enter the bile duct of the caudate lobe, resulting in implantable metastasis of the hepatic caudal lobe.^[42]^ Therefore, it is believed that the surgical treatment of HCCA should be combined with resection of the caudate lobe.^[38]^ However, removal of the caudate lobe increases the complexity of the procedure; therefore, several studies^[34,35]^ have proposed that caudate lobe resection should be selective.

In our study, the radical cure rate was 84.81% in the caudate lobe resection group and 60.31% in the caudate lobe preserved group. After combining 6 articles, the OR value was 3.88 (95% CI: 2.18–6.90), which indicated that the probability of radical resection combined hepatic caudate lobe resection was significantly higher than that in the caudate lobe preserved group. However, due to significant heterogeneity in the literature (I^2^ = 65.9%, *P* = .012), this conclusion still needs to be confirmed in well-designed large-scale clinical trials.

In the caudate lobectomy survival group, combined HR values showed that the survival of HCCA patients treated with combined caudate lobe resection was better than that in the caudate lobe preserved group (HR 0.45, 95% CI 0.38–0.55). In a subgroup analysis, regardless of the value of HR derived directly or indirectly, the sum of HR values indicated that the survival of HCCA patients treated with combined caudate lobe resection was better than that in the caudate lobe preserved group. This finding showed the importance of caudate lobe resection in surgery of HCCA. However, there is a lack of comparison of different types of Bismuth–Corlette classification in the selected literature; thus, it is not possible to conclude that caudate lobe resection is suitable for different types of HCCA.

In this study, the postoperative complication rate was 36.34% in the caudate lobectomy group, whereas it was 47.92% in the caudate lobe preserved group. The postoperative mortality in the caudate lobectomy group was 4.51% compared with 4.76% in the caudate lobe preserved group. The combined OR values showed that the complication rate, the risk of biliary leakage and mortality did not increase by combining caudate lobe resection. Dinant, S.^[43]^ investigated the postoperative complications and mortality in patients with HCCA between 1998 and 2003 and found that combined caudate lobe resection did not increase these risks. The results of our study are consistent with the conclusions reported by Dinant, S. It may be because resection of the caudate lobe is performed on the basis of left hepatectomy or right hepatectomy. On the one hand, it can ensure the negative cutting edge of the proximal bile duct. On the other hand, after left or right hepatectomy, it is easier to expose and resect the caudate lobe. Although combined hepatic caudate lobectomy increases the difficulty of surgery, postoperative complications, such as liver failure, infection, and bleeding, and mortality are mainly related to hepatectomy.^[36]^

Among the 10 articles included, the decision of caudate lobectomy was based on the experience of surgeon^[11,14,29,33–36,38,39]^ or the results of frozen section.^[37]^ Further, this begs the question of whether the decision regarding performing a caudate resection may be reflective of a preoperative concern for more advanced or more aggressive disease. But direct comparison between those studies proves difficult because of the lack of standardization in surgeon experience with hepatic resections (hilar cholangiocarcinoma) as well as differences in annual volume among contributing centers.

There are several limitations in this meta-analysis. The inclusion of non-randomized control trials leads to potential confounding bias. Some of the studies included were reports of experience. The length of follow-up and study quality varied across the studies. Also, complications were grouped together in majority of trials.

Despite these limitations, this meta-analysis has clinical value. The present analysis confirmed that combined caudate lobectomy significantly improves the radical cure rate and the survival time after the HCCA operation, and the postoperative complications and mortality are not increased. It is stand for a suggest that caudate lobectomy is necessary. However, more RCTs are warranted to further evaluated the mortality, using unification tools such as Clavien Dindo classification to evaluate the complications after Caudate lobectomy are recommend.

## Acknowledgments

The authors thank LetPub (www.letpub.com) for its linguistic assistance during the preparation of this manuscript.

## Author contributions

**Data curation:** Ming Yang.

**Formal analysis:** Ming Yang, Weiwei Li, Jianhua Chen, Miaohang Cui, Jinlong Liu.

**Investigation:** Jinlong Liu.

**Methodology:** Ming Yang, Jinlong Liu.

**Software:** Ming Yang.

**Supervision:** Jinlong Liu.

**Visualization:** Ming Yang.

**Writing – original draft:** Ming Yang.

**Writing – review & editing:** Ming Yang, Jinlong Liu.
